# LINC00205 promotes malignancy in lung cancer by recruiting FUS and stabilizing CSDE1

**DOI:** 10.1042/BSR20190701

**Published:** 2020-10-05

**Authors:** Peng Xie, Yesong Guo

**Affiliations:** Department of Radiotherapy, Jiangsu Cancer Hospital, Jiangsu Institute of Cancer Research, The Affiliated Cancer Hospital of Nanjing Medical University, Nanjing, Jiangsu 210009, China

**Keywords:** CSDE1, FUS, LINC00205, lung cancer

## Abstract

Lung cancer (LC) is characterized by high morbidity and mortality. Numerous long noncoding RNAs (lncRNAs) have been reported to be involved in the initiation and progression of human cancers, including LC. Long intergenic non-protein coding RNA 205 (LINC00205) is identified as a novel lncRNA, which has only been unmasked to be a potential cancer promoter in hepatocellular carcinoma and pancreatic cancer. The biologic function and the molecular mechanism of LINC00205 in LC require to be investigated. In the present study, we observed the elevated expression of LINC00205 in LC tissues and cells through real-time quantitative PCR (RT-qPCR). Additionally, silencing LINC00205 inhibited LC cell growth and migration, but aggravated cell apoptosis. Moreover, we found that LINC00205 recruited FUS to maintain the mRNA stability of cold shock domain containing E1 (CSDE1) and therefore up-regulated CSDE1 expression in LC. Further, the effects of LINC00205 on LC cell proliferation, apoptosis and migration were all erased by CSDE1 overexpression. These findings demonstrated that LINC00205 facilitates malignant phenotypes in LC by recruiting FUS to stabilize CSDE1, suggesting LINC00205 as a potential target for LC therapy.

## Introduction

Lung cancer (LC) has been characterized as a malignancy with the highest mortality, with the 5-year survival rate of approximately 20% [1,2]. In the past decades, mounting efforts have been put into discovering new prognostic biomarkers and therapeutic targets for LC [3,4]. Nevertheless, the detailed molecular mechanism of LC carcinogenesis and tumor progression needs to be better understood.

Long noncoding RNAs (lncRNAs) are featured with none protein coding capacity and the length of more than 200 nucleotides [5,6]. Plenty of reports have unmasked the association between lncRNA dysregulation and cancer development. Wang et al. found that lncRNA EGFR-AS1 promotes cell growth and metastasis in renal cancer [7]. Shi et al. discovered that lncRNA ZNFX1-AS1 facilitates tumor progression and metastasis in colorectal cancer [8]. Shi et al. revealed lncRNA FAM83A-AS1 as a tumor promoter in LC [9]. Long intergenic non-protein coding RNA 205 (LINC00205) is located at chromosome 21, NC_000021.9, and it is also named as LOC642852. LINC00205 is an lncRNA with poor annotation. Cui et al. supported that LINC00205 overexpression contributes to poor prognosis of patients with hepatocellular cancer [10]. Giulietti et al. unveiled LINC00205 as a novel biomarker for pancreatic cancer [11]. Nevertheless, the role of LINC00205 in LC is still veiled.

Here we proposed to explore the association between LINC00205 and LC, as well as the potential regulatory mechanism of LINC00205 in LC.

## Materials and methods

### Tissue collection

Seventy tumor and paired non-tumor tissues were acquired from Jiangsu Cancer Hospital with the approval of the Ethics Committee of Jiangsu Cancer Hospital. No patients underwent any therapies prior to surgery, and all of them signed the written informed consents before the present study. All samples were sharply frozen in liquid nitrogen after operation and then preserved at −80°C until use.

### Cell lines

Four human LC cell lines (A549, H1299, H23 and H522) and one normal epithelial cell line derived from human bronchus (BEAS-2B) were obtained from Chinese Cell Bank of the Chinese Academy of Sciences (Shanghai, China). All the cells were incubated with Dulbecco’s modified Eagle’s medium (DMEM, Gibio, U.S.A.) supplemented with 10% fetal bovine serum (FBS, Gibco) in a 37°C incubator which was humidified and supplied with 5% CO_2_.

### Cell transfection

Short hairpin RNAs (shRNAs) used to silence LINC00205 (sh-LINC00205-1, sh-LINC00205-2, sh-LINC00205-3) and the negative control (sh-NC) were synthesized by GenePharma (Shanghai, China). In addition, pcDNA3.1 vectors containing the full sequence of FUS or cold shock domain containing E1 (CSDE1) to up-regulate FUS or CSDE1 were provided by GenePharma (Shanghai, China), and the empty vectors were used as the corresponding negative controls. Forty-eight hours of transfection was accomplished using Lipofectamine® 2000 (Invitrogen, MA, U.S.A.) on the basis of the manufacturer’s instructions. The sequences used in cell transfection were shown in Supplementary Table S1.

### Real-time quantitative PCR

Isolation of total RNA was conducted using TRIzol solution (Invitrogen), followed by reverse transcription of isolated RNA with PrimeScript™ RT reagent kit (Takara, Dalian, China). Real-time quantitative PCR (RT-qPCR) was performed with the help of SYBR Premix Ex Taq II kit based on ABI 7500 Fluorescent Quantitative PCR system (Applied Biosystems Life Technologies, U.S.A.). The relative expression of LINC00205, FUS and CSDE1 was calculated by 2^−ΔΔ*C*_t_^ method and normalized to GAPDH. The primers utilized were:

GADPH, forward: 5′-TATGATGATATCAAGAGGGTAGT-3′,
reverse: 5′-TGTATCCAAACTCATTGTCATAC-3′.
LINC00205: forward, 5′-GGCTTTTGTGCCTGGAAGTG-3′,
reverse, 5′-GGGAAGTTCTGAGCTGGCAT-3′.
FUS: forward, 5′-GCCAAGATCAATCCTCCATGAGTAGTG-3′,
reverse, 5′-TCCACGGTCCTGCTGTCCATAG-3′.
CSDE1: forward, 5′-TGTACCGGGCAATCTCACAG-3′,
reverse, 5′-TGTCCCTCTTCTCACCCACT-3′.

### Western blot analysis

Proteins were obtained after cell lysis with RIPA and isolated with sodium dodecyl sulfate/polyacrylamide gel electrophoresis (SDS/PAGE) at the indicated concentration. After that, proteins were then preserved on PVDF membranes, which were further processed with incubation of primary and secondary antibodies in succession. Primary antibodies were as follows: anti-cleaved PARP (#5625, Cell Signaling Technology), anti-PARP (#9542, Cell Signaling Technology), anti-cleaved caspase-3 (#9661, Cell Signaling Technology), anti-caspase-3 (#9662, Cell Signaling Technology), anti-FUS (1:2000; Proteintech), anti-CSDE1 (1:1000, Abcam), anti-GAPDH (1:2000, Abcam) and anti-Tubulin (ab7291, Abcam). The Western blots were detected using ECL Detection Systems (Thermo Scientific, MA, U.S.A.) and imaged by Chemi Imager 5500 V2.03 software.

### Cell proliferation assay

For cell viability, Cell Counting Kit-8 (CCK-8, Beyotime Institute of Biotechnology, China) assay was carried out. In brief, cells at the concentration of 1000 cells/well were preserved in 96-well plates and incubated with indicated culture medium for 24 h. A total of 10 μl CCK-8 solution was added to each well and further incubated cells for 2 h at 37°C. The optical density was obtained using a SpectraMax M5 microplate reader (Molecular Devices, CA, U.S.A.) at 450 nm.

5-ethynyl-2′-deoxyuridine (EdU) assay was also performed to evaluate cell proliferation. After 2 h of incubation with EdU solution, cells were fixed by 4% paraformaldehyde. Following staining with Cell-Light™ EdU Apollo®488 In Vitro Imaging Kit (RioBio, China), cells with positive EdU were captured by fluorescence microscopy.

### Flow cytometry assay

Cell apoptosis was assessed by use of Annexin-V fluorescein isothiocyanate (FITC)/propidium iodide (PI) double-staining kit (Abcam, Cambridge, U.K.) in line with the instructions of manufacturer. The apoptotic cells were determined using BD FACSCalibur flow cytometry (BD Biosciences, San Jose, CA).

### Cell migration assay

For cell migration evaluation, transwell assay was utilized. Briefly, cells were seeded in the upper chamber containing serum-free medium. On the other hand, the lower chamber was supplied with culture medium plus 10% FBS as a chemoattractant. After incubation for 24 h, cells migrated from the upper chamber to the lower chamber were immobilized by 4% paraformaldehyde. After staining with 1% Crystal Violet, the migrated cells were counted under a light microscope.

### Cellular fractionation assay

On the basis of the manufacturer’s protocols, PARIS™ kit (Ambion, AM1921) was utilized to separate nuclear and cytoplasmic RNAs. Then, these RNAs were analyzed via RT-qPCR. GAPDH served as the cytoplasmic control while U2 as the nuclear control.

### Caspase-3 activity assay

Cells were lysed with RIPA buffer (Beyotime). The supernatants were acquired after centrifugation and placed on a 96-well plate. Reaction buffer plus caspase-3 substrate (Ac-DEVD-pNA) was added to each well for incubation at the temperature of 37°C. Four hours later, the optical density (OD) was evaluated at 405 nm by a microplate reader.

### RNA pull-down assay

LINC00205-sense, LINC00205-antisense, and the negative control nonsense sequences were biotinylated with biotinylated RNA labeling mix (Roche) to obtain Bio-LINC00205, Bio-LINC00205-antisense and Bio-NC. After that, these biotin-labeled RNAs were mixed with whole-cell lysates extracted from A549 and H1299 cells. Streptavidin agarose beads (Invitrogen) were utilized to pull down the complexes. RNAs in these complexes were assessed by RT-PCR analysis. Input was the positive control.

### RNA immunoprecipitation assay

RNA immunoprecipitation (RIP) experiments were conducted under the help of a Magna RIP RNA-Binding Protein Immunoprecipitation Kit (Millipore). Cells were harvested and lysed for RIP process with the antibody against FUS or IgG (negative control). Input served as the positive control. RNA precipitated in indicated groups was evaluated by RT-qPCR.

### Statistical analysis

Data analysis was carried out by the Statistical Package for the Social Sciences (SPSS 16.0; SPSS Inc., Chicago, IL). Student’s *t* test was utilized for the difference between two groups, and one-way ANOVA was applied for analyzing data from more than two groups. Statistical significance was defined as *P*-value <0.05.

## Results

### LINC00205 is up-regulated in LC cells and contributes to LC progression

**Figure 1:**
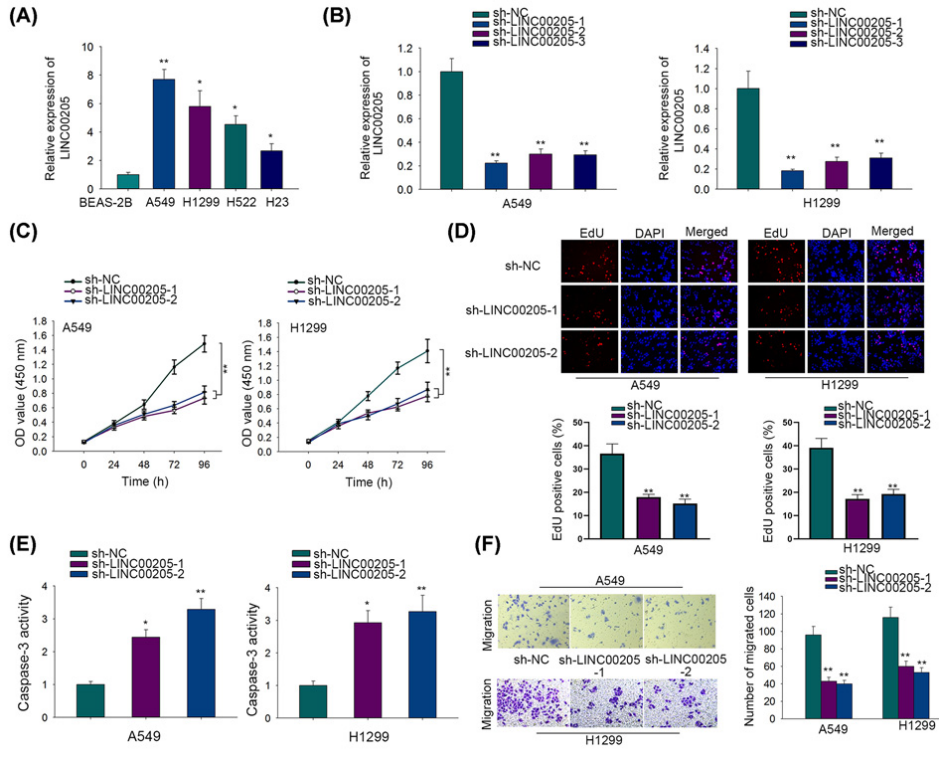
LINC00205 was up-regulated in LC cell lines and contributed to malignant processes in LC cells (**A**) Expression levels of LINC00205 in four LC cell lines (A549, H1299, H23, H522) and one normal BEAS-2B cells were determined by RT-qPCR. (**B**) The expression level of LINC00205 were evaluated by RT-qPCR in cells transfected with indicated shRNAs (sh-NC, sh-LINC00205-1, sh-LINC00205-2 or sh-LINC00205-3). (**C**) CCK-8 assay was conducted to evaluate cell viability. (**D**) EdU assay was carried out to evaluate cell proliferation. (**E**) Caspase-3 activity assay was performed for cell apoptosis evaluation. (**F**) Cell migration was determined by transwell assay. The assays above were all carried out at least three times. *P<0.05, **P<0.01.

First, we analyzed the expression pattern of LINC00205 in LC. The results of RT-qPCR elucidated that LC tissues expressed higher level of LINC00205 than paired non-tumor tissues (Supplementary Figure S1A). Besides, LINC00205 expression was strongly increased in LC cells compared with that in normal BEAS-2B cells (Figure 1A). Next we transfected shRNAs targeting LINC00205 (sh-LINC00205-1, sh-LINC00205-2 and sh-LINC00205-3) into A549 and H1299 cells, and discovered markedly reduced LINC00205 level in these two cells after transfections (Figure 1B). Considering the better silencing efficiencies of sh-LINC00205-1 and sh-LINC00205-2, cells transfected with them were applied in the next loss-of-function assays. Interestingly, inhibiting LINC00205 significantly weakened cell viability, as showed by CCK-8 assay (Figure 1C). Consistently, EdU assay indicated that cell proliferation was much inhibited after LINC00205 knockdown (Figure 1D). By contrast, flow cytometry analysis indicated that LINC00205 depletion resulted in accelerated apoptosis in LC cells (Supplementary Figure S1B). Moreover, caspase-3 activity assay implied that the activity of caspase-3, a marker of cell apoptosis, was significantly strengthened after LINC00205 silence (Figure 1E). Additionally, we also detected augmented levels of cleaved RAPR and caspase-3 proteins in LINC00205-inhibited LC cells (Supplementary Figure S1C). Transwell assay demonstrated that down-regulating LINC00205 apparently hindered cell migration (Figure 1F). Hence, we concluded that LINC00205 is up-regulated in LC cells and contributes to malignancy in LC.

### LINC00205 interacts with FUS in LC

**Figure 2:**
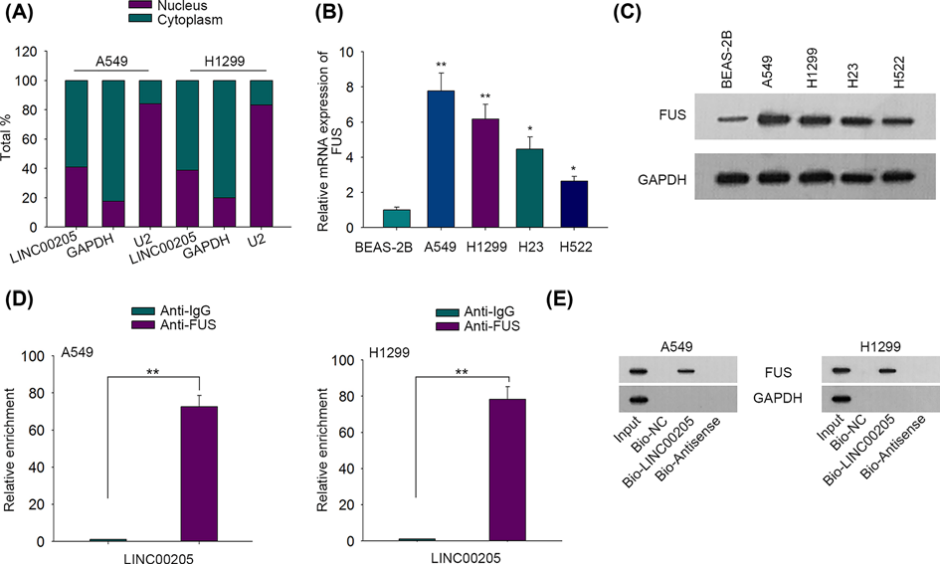
LINC00205 interacted with FUS in LC (**A**) Nuclear/cytoplasmic fractionation assay was used for detecting the localization of LINC00205 in A549 and H1299 cells. (**B**) The expression of FUS in LC cells and BEAS-2B cells was measured by RT-qPCR. (**C**) The protein levels of FUS in LC cells and BEAS-2Bcells were measured by Western blot. (**D**) RIP assay was carried out to measure the interaction between LINC00205 and FUS protein in LC cells. (**E**) RNA pull down followed by Western blot validated the interaction of LINC00205 with FUS protein in LC cells. The assays above were all carried out at least three times. *P<0.05, **P<0.01.

Hitherto, it has been validated that lncRNAs may participate in the regulation of tumor progression through interacting with certain proteins [12,13]. Bioinformatics from starBase2 (http://starbase.sysu.edu.cn/index.php) predicted FUS as a potential candidate which was capable of binding to LINC00205 with district stringency. Since FUS was recognized as an RNA-binding protein (RBP) and lncRNAs generally interact with RBPs in cytoplasm, we then wondered whether LINC00205 could interact with FUS in LC. Subcellular fractionation discovered that LINC00205 was mainly located in the cytoplasm of LC cells (Figure 2A), indicating the binding potential of LINC00205 with FUS in LC. Then we explored FUS expression in LC. The results of RT-qPCR indicated that FUS was overexpressed in LC cells relative to normal BEAS-2B cells (Figure 2B). Similarly, Western blot also identified the remarkably up-regulated protein level of FUS in LC cells compared with normal controls (Figure 2C). Afterward, RIP assay elucidated that LINC00205 was abundantly precipitated by anti-FUS (Figure 2D). In addition, RNA pull down further confirmed the direct interaction between LINC00205 and FUS, since FUS bands could only be observed in Input and Bio-LINC00205 groups (Figure 2E). In summary, LINC00205 interacts with FUS in LC.

### LINC00205 binds to FUS and therefore maintains mRNA stability of CSDE1

**Figure 3:**
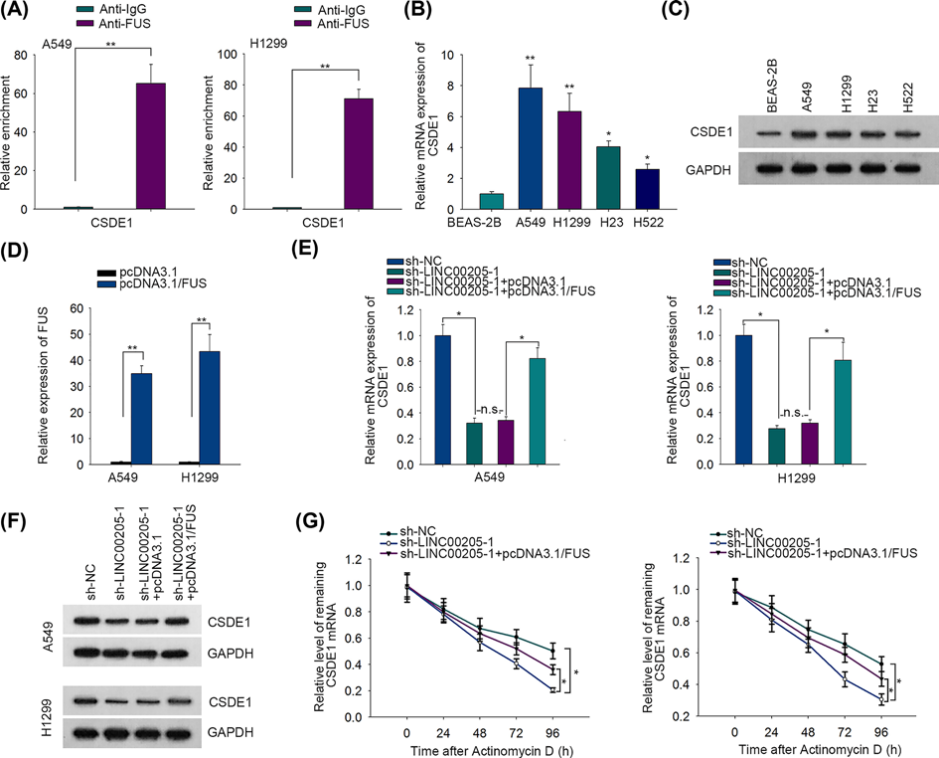
LINC00205 recruited FUS to maintain the mRNA stability of CSDE1 in LC (**A**) RIP assay determined the binding of CSDE1 mRNA to FUS or IgG in LC cells. (**B**) The expression of CSDE1 in LC cells was measured by RT-qPCR. (**C**) The protein level of CSDE1 in LC cells was measured by Western blot. (**D**) The expression levels of FUS were evaluated using RT-qPCR in A549 and H1299 cells transfected with pcDNA3.1/FUS or the empty vector. (**E,F**) The mRNA and protein levels of CSDE1 were respectively evaluated by RT-qPCR and Western blot in cells transfected with sh-NC, sh-LINC00205, sh-LINC00205-1+pcDNA3.1 or sh-LINC00205-1+pcDNA3.1/FUS. (**G**) RT-qPCR analyzed CSDE1 expression in LC cells after actinomycin D treatment for indicated times. The assays above were all carried out at least three times. *P<0.05, **P<0.01, n.s. meant no significance.

RBPs, including FUS, have been featured with the ability of maintaining mRNA stability [14,15]. Prediction from starBase2 indicated that CSDE1 was a potent gene whose mRNA was able to interact with FUS. Subsequent RIP assay confirmed such prediction, as CSDE1 mRNA was evidently precipitated by anti-FUS (Figure 3A). Then we detected the expression of CSDE1 by performing RT-qPCR and Western blot, and the results manifested the elevated mRNA and protein levels of CSDE1 in LC cells in comparison with control BEAS-2B cells (Figure 3B,C). Thereafter, we validated that the expression of FUS in 1549 and H1299 cells was markedly increased after transfecting with pcDNA3.1/FUS (Figure 3D). Moreover, it was revealed that the mRNA and protein levels of CSDE1 were both declined by depleted LINC00205, while such decline effect could be reversed after further overexpressing FUS (Figure 3E,F). More importantly, we unveiled that silencing LINC00205 significantly shortened the half-life of CSDE1 mRNA, whereas FUS overexpression partly rescued the facilitating impact of LINC00205 depletion on CSDE1 degradation (Figure 3G). Based on these findings, the conclusion could be drawn that LINC00205 binds to FUS to enhance mRNA stability of CSDE1.

### LINC00205 contributes to LC progression via up-regulating CSDE1

**Figure 4:**
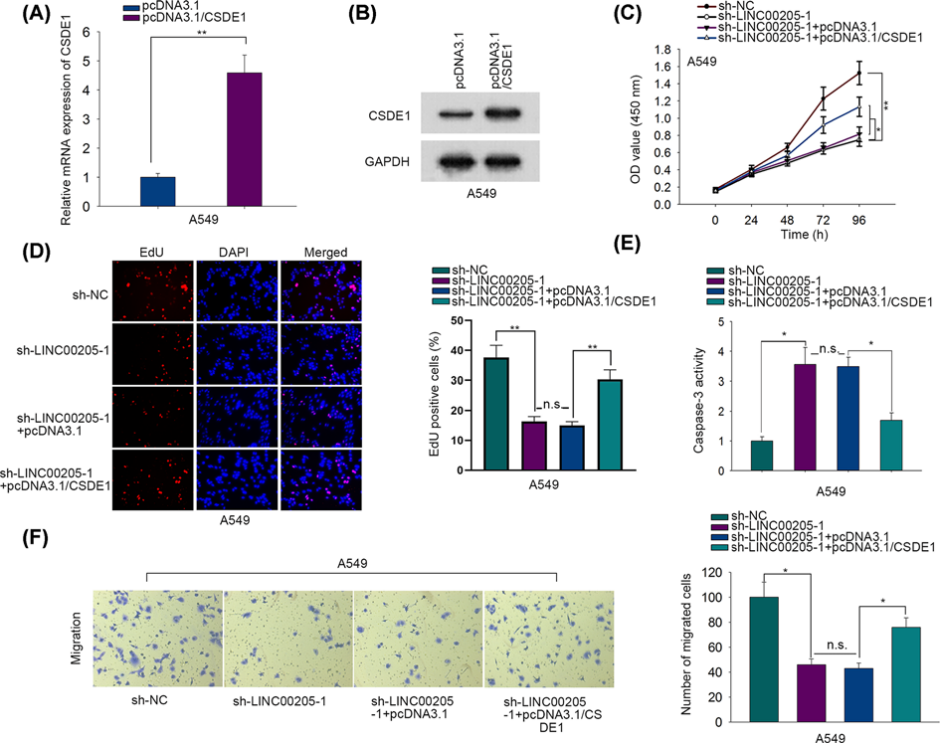
LINC00205 contributed to LC progression via up-regulating CSDE1 (**A,B**) The mRNA and protein levels of CSDE1 were correspondingly examined by RT-qPCR and Western blot in cells treated with pcDNA3.1/CSDE1 or thee empty vector. (**C**) Cell viability was determined by CCK-8 assay. (**D**) Cell proliferation was evaluated with EdU assay. (**E**) Caspase-3 activity assay was conducted to evaluate cell apoptosis. (**F**) Cell migration was detected by transwell assay. The assays above were all carried out at least three times. *P<0.05, **P<0.01, n.s. meant no significance.

To confirm whether LINC00205 facilitated malignant behaviors of LC cells through regulating CSDE1, we performed rescue assays. Prior to that, we proved that CSDE1 expression levels were apparently elevated after transfecting with pcDNA3.1/CSDE1 (Figure 4A,B). Further, we uncovered that the impaired growth of A549 cells in response to LINC00205 silence was partly recovered when co-transfected with pcDNA3.1/CSDE1 (Figure 4C,D). Also, overexpressing CSDE1 abrogated the enhancing effect of LINC00205 down-regulation on caspase-3 activity (Figure 4E). In addition, CSDE1 up-regulation had an erasing impact on the weakening function of LINC00205 silence in cell migration (Figure 4F). Meanwhile, we also found similar phenomena in H1299 cells, evidenced by enhanced expression of CSDE1 counteracted the influence of LINC00205 suppression on H1299 cell proliferation, apoptosis and migration (Supplementary Figure S1D–I). In conclusion, LINC00205 contributes to LC progression via up-regulating CSDE1.

## Discussion

LC has been characterized as the malignancy with the highest incidence and lethality rates [2]. Even though a large number of researchers have spared no effort to find new therapies, the overall survival rate of LC patients remains far from satisfactory [16].

In recent years, the role of lncRNAs in tumor initiation and progression has drawn much attention [17–19]. LINC00205 has been annotated as an oncogenic lncRNA in pancreatic cancer and hepatocellular carcinoma [10,11]. In the current study, we identified LINC00205 as notably overexpressed in LC tissues and cells. We also observed that silencing LINC00205 suppressed cell growth and migration in LC.

FUS/TLS, encoded by the *FUS* gene, belongs to the RBP family. A great number of evidences have revealed the tumor promoting role of FUS in carcinomas. For example, Tornin et al. discovered that FUS-CHOP promotes cell invasion in myxoid liposarcoma [20]. He et al. found that FUS regulates angiogenesis in glioma [21]. In our study, FUS was confirmed to be up-regulated in LC cells and could interact with LINC00205.

RBPs possess the ability to maintain mRNA stability and abundance. This is a much more efficient way to maximum cell survival through stabilizing mRNAs rather than producing proteins [22]. CSDE1, which has already been validated to be involved in several cancers [23,24], was predicted as a possible candidate mRNA to interact with FUS through bioinformatics in present work. Also, we observed that CSDE1 was greatly up-regulated in LC cells. Moreover, we disclosed that LINC00205 recruited FUS to maintain mRNA stability of CSDE1. At last, a series of rescue assays indicated that LINC00205 contributed to LC cell growth and migration via up-regulating CSDE1.

To draw a conclusion, we discovered that LINC00205 aggravates LC progression through recruiting FUS and therefore stabilizing CSDE1. Our findings suggest that LINC00205 might serve as a potential therapeutic target for LC.

## Supplementary Material

Supplementary Figure S1 and Table S1

## Abbreviations

CCK-8: Cell Counting Kit-8
CSDE1: cold shock domain containing E1
EdU: 5-ethynyl-2′-deoxyuridine
FUS: FUS RNA binding protein
GAPDH: glyceraldehyde-3-phosphate dehydrogenase
LC: lung cancer
lncRNA: long noncoding RNA
LINC00205: long intergenic non-protein coding RNA 205
PARP: poly ADP-ribose polymerase
RBP: RNA-binding protein
RIP: RNA immunoprecipitation
RT-qPCR: real-time quantitative PCR
shRNA: short hairpin RNA
SPSS: Statistical Package for the Social Sciences
