# Supplementary material for: LINC00205 promotes malignancy in lung cancer by recruiting FUS and stabilizing CSDE1
Source: Biosci Rep. 2020 Oct 5;40(10):BSR20190701. doi: 10.1042/BSR20190701 (PMC7536328; doi:10.1042/BSR20190701)
Supplement: Supplementary Figure S1 and Table S1 [file BSR-2019-0701_supp.pdf]

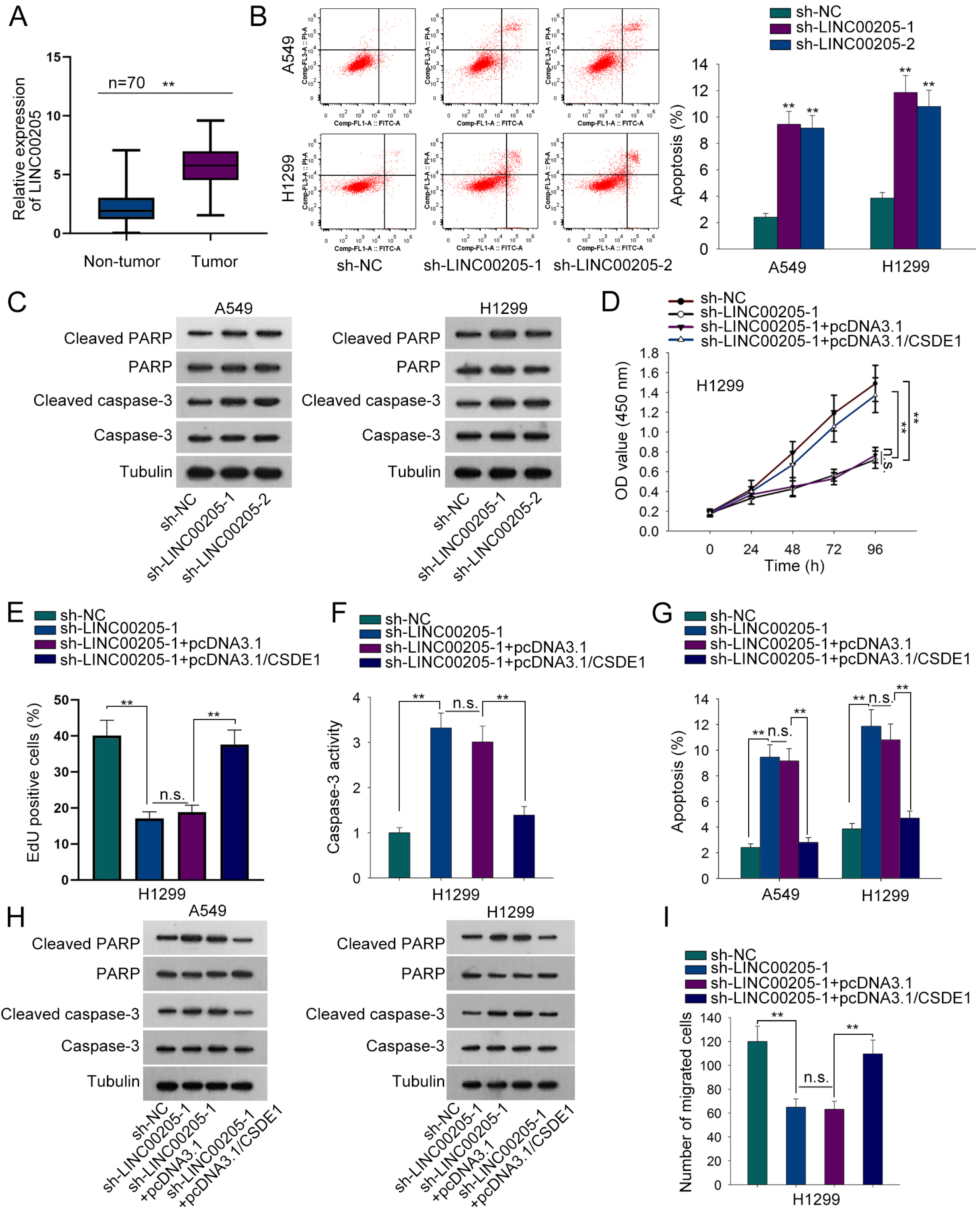
A
n=70 **
Relative expression of LINC00205
Non-tumor
Tumor
B
A549
H1299
sh-NC
sh-LINC00205-1
sh-LINC00205-2
Apoptosis (%)
C
Cleaved PARP
PARP
Cleaved caspase-3
Caspase-3
Tubulin
D
sh-LINC00205-1+pcDNA3.1
sh-LINC00205-1+pcDNA3.1/CSDE1
OD value (450 nm)
Time (h)
n.s.
E
EdU positive cells (%)
F
Caspase-3 activity
G
H
I
Number of migrated cells

**Supplementary Figure S1.** (A) RT-qPCR tested the expression of LINC00205 in 70 LC tissues and matched non-tumor tissues. (B) The apoptosis of LC cells was determined by flow cytometry analysis. (C) western blot evaluated the levels of proteins related to apoptosis, including cleaved PARP, cleaved caspase-3 and the total PARP and caspase-3, in LC cells with or without LINC00205 silence. (D-I) The viability, proliferation, apoptosis and migration of H1299 cells under indicated contexts was examined through conducting CCK-8 assay, EdU assay, caspase-3 activity detection assay, flow cytometry analysis, western blot analysis and transwell assay. The assays above were all carried out at least three times. $^{**}P < 0.01$, n.s. meant no significance.

# **Supplementary Table S1.** Sequences used in cell transfection.

| Plasmids | Sequences (5'-3') |
|---|---|
| sh-NC | CCGGAAAAGTTATTTGACCCAGAGTCTCGAGACTCTGGGTCAAATAACTTTTTTTTTG |
| sh-LINC00205-1 | CCGGTGGTCTTTGAAAGACACTTAACTCGAGTTAAGTGTCTTTCAAAGACCATTTTTG |
| sh-LINC00205-2 | CCGGTGGGGAAAATACTCAGAATAACTCGAGTTATTCTGAGTATTTTCCCCATTTTTG |
| sh-LINC00205-3 | CCGGTCCAGTAATTTGACATAAAGACTCGAGTCTTTATGTCAAATTACTGGATTTTTG |
| pcDNA3.1/FUS | ACTTAAGCTTCGACGCAGGAGGCGGGGCTGCTCAGTCCTCCAGGCGTCGGTACTCAGCGGTGTTGGAACT |
| | TCGTTGCTTGCTTGCCTGTGCGCGCGTGCGCGGACATGGCCTCAAACGATTATACCCAACAAGCAACCCA |
| | AAGCTATGGGGCCTACCCCACCCAGCCCGGGCAGGGCTATTCCCAGCAGAGCAGTCAGCCCTACGGACAG |
| | CAGAGTTACAGTGGTTATAGCCAGTCCACGGACACTTCAGGCTATGGCCAGAGCAGCTATTCTTCTTATG |
| | GCCAGAGCCAGAACAGCTATGGAACTCAGTCAACTCCCCAGGGATATGGCTCGACTGGCGGCTATGGCAG |
| | TAGCCAGAGCTCCCAATCGTCTTACGGGCAGCAGTCCTCCTACCCTGGCTATGGCCAGCAGCCAGCTCCC |
| | AGCAGCACCTCGGGAAGTTACGGTAGCAGTTCTCAGAGCAGCAGCTATGGGCAGCCCCAGAGTGGGAGCT |
| | ACAGCCAGCAGCCTAGCTATGGTGGACAGCAGCAAAGCTATGGACAGCAGCAAAGCTATAATCCCCCTCA |
| | GGGCTATGGACAGCAGAACCAGTACAACAGCAGCAGTGGTGGTGGAGGTGGAGGTGGAGGTGGAGGTAAC |
| | TATGGCCAAGATCAATCCTCCATGAGTAGTGGTGGTGGCAGTGGTGGCGGTTATGGCAATCAAGACCAGA |
| | GTGGTGGAGGTGGCAGCGGTGGCTATGGACAGCAGGACCGTGGAGGCCGCGGCAGGGGTGGCAGTGGTGG |
| | CGGCGGCGGCGGCGGCGGTGGTGGTTACAACCGCAGCAGTGGTGGCTATGAACCCAGAGGTCGTGGAGGT |
| | GGCCGTGGAGGCAGAGGTGGCATGGGCGGAAGTGACCGTGGTGGCTTCAATAAATTTGGTGGCCCTCGGG |
| | ACCAAGGATCACGTCATGACTCCGAACAGGATAATTCAGACAACAACACCATCTTTGTGCAAGGCCTGGG |
| | TGAGAATGTTACAATTGAGTCTGTGGCTGATTACTTCAAGCAGATTGGTATTATTAAGACAAACAAGAAA |
| | ACGGGACAGCCCATGATTAATTTGTACACAGACAGGGAAACTGGCAAGCTGAAGGGAGAGGCAACGGTCT |
| | CTTTTGATGACCCACCTTCAGCTAAAGCAGCTATTGACTGGTTTGATGGTAAAGAATTCTCCGGAAATCC |
| | TATCAAGGTCTCATTTGCTACTCGCCGGGCAGACTTTAATCGGGGTGGTGGCAATGGTCGTGGAGGCCGA |
| | GGGCGAGGAGGACCCATGGGCCGTGGAGGCTATGGAGGTGGTGGCAGTGGTGGTGGTGGCCGAGGAGGAT |
| | TTCCCAGTGGAGGTGGTGGCGGTGGAGGACAGCAGCGAGCTGGTGACTGGAAGTGTCCTAATCCCACCTG |
| | TGAGAATATGAACTTCTCTTGGAGGAATGAATGCAACCAGTGTAAGGCCCCTAAACCAGATGGCCCAGGA |
| | GGGGGACCAGGTGGCTCTCACATGGGGGGTAACTACGGGGATGATCGTCGTGGTGGCAGAGGAGGCTATG |
| | ATCGAGGCGGCTACCGGGGCCGCGGCGGGGACCGTGGAGGCTTCCGAGGGGGCCGGGGTGGTGGGGACAG |
| | AGGTGGCTTTGGCCCTGGCAAGATGGATTCCAGGGGTGAGCACAGACAGGATCGCAGGGAGAGGCCGTAT |
| | TAATTAGCCTGGCTCCCCAGGTTCTGGAACAGCTTTTTGTCCTGTACCCAGTGTTACCCTCGTTATTTTG |
| | TAACCTTCCAATTCCTGATCACCCAAGGGTTTTTTTGTGTCGGACTATGTAATTGTAACTATACCTCTGG |
| | TTCCCATTAAAAGTGACCATTTTAGTTAAATTTTGTTCCTCTTCCCCCTTTTCACTTTCCTGGAAGATCG |
| | ATGTCCCGATCAGGAAGGTAGAGAGTTTTCCTGTTCAGATTACCCTGCCCAGCAGGAACTGGAATACAGT |
| | GTTCGGGGAGAAGGCCAAATGATATCCTTGAGAGCAGAGATTAAACTTTTCTGTCATGGGGAAAGTTGGT |
| | GTATAAATGAGAAATGAAGAACATGGGATGTCATGAGTGTTGGCCTAAATTTGCCCAGCTATGGGGAATT |
| | TTTCCTTTACCACATTTATTTGCATACTGGTCTTAGTTTATTTGCAGCAGTTTATCCCTTTTTAAGAACT |
| | CTTTGATCTTTTGGCCCTTTTAATGGTGAGGCTCAAACAAACTACATTTAAATGGGGCAGTATTCAGATT |
| | TGACCATGGTGGAGAGCGCTTAGCCACTCTGGGTCTTTCACAGGAAGGAGAGTAACTGAGTGCTGCAGGA |
| | GTTTGTGGAGTGGAGTCAGGATCTAGGAGGTGAGTGACTCCCTTCCTAGCTGCCCTGGTGAACAGCGCTT |
| | GGGTAGATACCTGCTATAAGGAGACTGGTCTGGCTGGGTTACTTTCACATCCTGCCTGTACTCAGAGGGC |
| | TTGAGGTCATTGACATTATGAGATTTTAGGCTTGATCCCTTTTTGATTGGAGGGTGGAAGGCCCTCCTAA |
| | GGGAATGATAAGTGATAAGAGGGGGAAGGGGTTGCAGCCAATGAGTTAAAACCTTAGAGCAGTGCTCCTC |
| | AGCCTCTTACCATGTGGTTGTAAACTTGCACGTACCTGCCAACCAGTTATTTAGCATGCTTTTTATTTTA |
| | GTTACACAGAGCGTAACATTAACCCAAGAGCAGAAAGGTTTTATTTACAGGGTTTTCGAACTTGGTTTGT |
| | AAGACAGCTGCCATCACAAGCATAGCTTACAAATGTGCTGGGGACCCCTAATTGGGAAGTGCTTTCCTCT |
| | CAAATTTTTATTTTTTATTTTTAGAGACAGAGTCTTGCTCTGTCATCCAGGCTGGAGTGCAGTGGCGTGA |
| | TCTCGGCTCACTGTAGCCTCTGCCTCCTGAGTTTAAGCGATTCTCCTGCCTCAGGAGAATCCCAGCTTCT |
| | GAGTAGCTGAGACTACAGGCGTGGGCCACCATGCCCACCTAGTTTTTGCATTTTTAGTGGAGGTGTGGTT |
| | TCACTGTGTTGGCCAGGCTGGTCTCTTAACTCCTGACCTCAGGTGATCCACCTGCCTTGGCCTCCCAAAG |
| | TGCTGGAATTACAGGCATGAGCCGCTGCATCTGGCCATCCTCTCAAATTTTCAAGTGTTCCACAAGTATG |
| | TTCTCTACTGAAGAGTTGCTGCATCCTTGAATCTTGGGTGATTTGAGGCACAGAAACTATGACTTTATTT |
| | TTTGAGATGGAGTTTTGCTCTTGTTGCCCAGGCTGGAGTGCAATGGCACGTTTTTCGGCTTACCGCAACT |
| | GCCGCCTCCTGGGTTCAAGCGATAGCTGGGATTACAGGCATGCGCCACCATGCCCAGCTAATTTATTTGT |
| | ATTTTTAGTAGAGACGGGGTTTCTCCATGTTGGTCAGGCTGGTCTCGAACTCCCGACCTCAGGTGATGTG |
| | CACACCTCAGCCTCCCAATAAACCATGACTTTTAAGAGGAATAGCAGGTTTACTTCCCCTGCCAGCATTG |
| | GGGTGCTCTCTAAGCAACAGTAGGCGGAGAGTGGTCTGGCGTATTAAAAACAAAGGATCGTCAAGTGGGC |
| | CTTCCCAGGCATTGCTTTGACTTAGTACATGTAGAGGATGTGGCAGTTCTCTCCGTCCCTGCCACTGCTG |
| | GTTTCTTTGTTAAATGTTTAGTTGAAATGGCCTGATACGATATTTGAGTAGTTCACTGTTGGTGCTTTGC |
| | CTAGCAGGATTCTAATCTTGCTTTGGTTGTGGTCCCCTGATGCCCTCCTGTTAGGAGTGGAGGAGGTCGA |
| | AGCTCCTTGTAAGATATGATTACTGGGACCATTAGTGTCAAGTTCCTGTGTCCTTCAAATGGCATATGTG |
| | ATTGGCCTTGACCTTAAAAGGAAATAGGGTCCCAGGTGACTGTTTAGTGGGTAGGTCCAGTTTGGGGGGA |
| | TCTTCCAGGAGAATGGATAGAGACACCTAGCAGCAGAGAGAACATTGGTGCCTCTCAAGCCAACCTCCCA |
| | CCTCAGCCTCCCAAGTAGCTGGGACTACAGGTGCTTCCTCGCTACCACACCTGGGTAATTTTTTTTTTAA |
| | TTACTTTTTTTTTTTTAAGAAACAGGGTTTCACTGGGTTGCCCAGGCTGGTCTTGAACTGGCCTCAAGTG |
| | ACCTGCCTGCCTCAGCCTCCCAAAGTGCTGGGATCACAGGTGTGACCCACTGCGTTTGGCCAGAATACTC |
| | TATTCTTACTGAATGATTGAAATCTGTCTTGAAGCATTAGGTGTCCCATTTTTGTGAGTTGGAATTGGGA |
| | CAGGCTAAGTAGGAAGTGAGGAGGGTGGGGAGAGCTGTGCTGTAGGTCTGTTTGTCCCTTCCTTGATGTA |
| | GCCTTCAGTTAGCCCTTTCAGCTTTTTCCCCATCTTGTGCCGGGCCTTCCTGGGTTTCAGTACTTGGAT |
| | GTAGGGCTGCAGTTATGTCAGTGGTGGGTAGATTGACCAGGAATTAAGGTCTAGGGTCCAGCCCATGTGA |
| | GACTTGACTCACTGATCTACCTTTAGGCATGTCTTCCTTCCAGTCTCATCCTTTTTAAATTTTTTTTTTT |
| | TTTTTGAGACGGTCTCACTCTCACCCAGGCTGGATTGCAGTGGTGTGATCTCGACCAACTGCAACCTCTG |
| | CCTCCCACCCGCAAGCTATCTGCCCACCTCAGCCTCTGGAGTAGCTGGGACGGGACTACAGGCACCTGCC |
| | ACCATGACTGGCTAATTTTTTTTTGTATTTTTGTGGAGATGGGGTCTTGCCATGTTGCTCAGGCTGGTC |
| | TGGTCTCAAAACTGCTCTGGGCTCAAGTGATTGTCCCACTTTGGCCTCCCAGAGTGCTGGGATTAAGGTG |
| | TGAGATACTGTGTCCGGCTATGAAAATTTTATTTTTAATTAACTTGTATATATTTATGGGGTACAATGTC |
| | CTATTTCTGTACATGTACACATTGTGGAATCAAATCAGGCTAATATATCCATCACTTCATATCATTAGCA |
| | TGAATGAGAACATACAAAGCCACTCTTAGAAAATTTTGAAATTTATGTTATTTCAGCCCTTTTATGCTGG |
| | AGGTTGCAAATGTTTTGTGAATAATCAGACCAAAAATAAAAACAAAAAATGATTGACTTCAGTCATTCAG |
| | TAAGAA |
| pcDNA3.1/CSDE1 | CCTACGTACCGGGCCGTGCTGCTTATGGCGGCGCTGGAGAGGGGGCGCTGAGCTGTTGGGTATGAAGTGT |
| | AACAGAACAGACTTTACCACCTGAAACTGCTGCTTCAAGTTCAGATCAGGCAAGGAACAAACCTCGTAAC |
| | AACTAACAAGACCAAAGAAGAGTACACTTAAGTTGAAGACACAACACTTGATCTGAAACAAGAAGTTTGT |
| | GCCTACTCAACAGCTTTGAAAGAGCACTTCCCAACGCTGCTAGTAGTCTTTGTTTTCTTCAGTGCTGTAC |
| | TGTGAGATTGCCCGGTACAGCAGCAGTTGTATTCTTTATTAGCTTGGTAGATCATTTTCTCTCGCTCTTT |
| | TTTTTAATACTAGCAACTTTCATCCTTTGAAACGTGTGCTGAAAAAGAAGAATCAGCAAATACTACTGAA |
| | AGTGCAATATTTGAGTATCACTGCGAGATGAGCTTTGATCCAAACCTTCTCCACAACAATGGACATAATG |
| | GGTACCCTAATGGTACTTCAGCAGCACTGCGTGAAACTGGGGTTATTGAAAAACTGTTAACCTCTTACGG |
| | ATTTATTCAGTGTTCAGAACGTCAAGCTAGACTTTTCTTCCACTGTTCACAGTATAATGGCAACCTGCAA |
| | GACTTAAAAGTAGGAGATGATGTTGAATTTGAAGTATCATCGGACCGACGGACTGGGAAACCCATTGCTG |
| | TTAAACTGGTGAAGATAAAACAAGAAATCCTCCCTGAAGAACGAATGAATGGACAAGTTGTGTGCGCTGT |
| | TCCTCACAACTTAGAGAGTAAATCTCCAGCTGCCCCGGGTCAGAGTCCAACAGGGAGTGTATGCTACGAA |
| | CGTAATGGGGAAGTGTTTTATCTGACTTACACCCCTGAAGATGTCGAAGGGAACGTTCAGCTGGAAACTG |
| | GAGATAAAATAAACTTTGTAATTGATAACAATAAACATACTGGTGCTGTAAGTGCTCGCAACATTATGCT |
| | GTTGAAAAAGAAACAAGCCCGCTGTCAGGGAGTAGTTTGTGCCATGAAGGAGGCATTTGGCTTTATTGAA |
| | AGAGGTGATGTTGTAAAAGAGATATTCTTTCACTATAGTGAATTTAAGGGTGACTTAGAAACCTTACAGC |
| | CTGGCGATGATGTGGAATTCACAATCAAGGACAGAAATGGTAAAGAAGTTGCAACAGATGTCAGACTATT |
| | GCCTCAAGGAACAGTCATTTTTGAAGATATCAGCATTGAACATTTTGAAGGAACTGTAACCAAAGTTATC |
| | CCAAAAGTACCCAGTAAAAACCAGAATGACCCATTGCCAGGACGCATCAAAGTTGACTTTGTGATCCCTA |
| | AAGAACTTCCCTTTGGAGACAAAGATACGAAATCCAAGGTGACCCTGCTGGAAGGTGACCATGTTAGGTT |
| | TAATATTTCAACAGACCGACGTGACAAATTAGAGCGAGCAACCAATATAGAAGTTCTGTCAAATACATTT |
| | CAGTTCACTAATGAAGCCCGAGAAATGGGTGTGATTGCTGCCATGAGAGATGGTTTTGGTTTCATCAAGT |
| | GTGTGGATCGTGATGTTCGTATGTTCTTCCACTTCAGTGAAATTCTGGATGGGAACCAGCTCCATATTGC |
| | AGATGAAGTAGAGTTTACTGTGGTTCCTGATATGCTCTCTGCTCAAAGAAATCATGCTATTAGGATTAAA |
| | AAACTTCCCAAGGGCACGGTTTCATTTCATTCCCATTCAGATCACCGTTTTCTGGGCACGGTAGAAAAAG |
| | AAGCCACTTTTTCCAATCCTAAAACCACTAGCCCAAATAAAGGCAAAGAGAAGGAGGCTGAGGATGGCAT |
| | TATTGCTTATGATGACTGTGGGGTGAAACTGACTATTGCTTTTCAAGCCAAGGATGTGGAAGGATCTACT |
| | TCTCCTCAAATAGGAGATAAGGTTGAATTTAGTATTAGTGACAAACAGAGGCCTGGACAGCAGGTTGCAA |
| | CTTGTGTGCGACTTTTAGGTCGTAATTCTAACTCCAAGAGGCTCTTGGGTTATGTGGCAACTCTGAAGGA |
| | TAATTTTGGATTTATTGAAACAGCCAATCATGATAAGGAAATCTTTTTCCATTACAGTGAGTTCTCTGGT |
| | GATGTTGATAGCCTGGAACTGGGGGACATGGTCGAGTATAGCTTGTCCAAAGGCAAAGGCAACAAAGTCA |
| | GTGCAGAAAAAGTGAACAAAACACACTCAGTGAATGGCATTACTGAGGAAGCTGATCCCACCATTTACTC |
| | TGGCAAAGTAATTCGCCCCCTGAGGAGTGTTGATCCAACACAGACTGAGTACCAAGGAATGATTGAGATT |
| | GTGGAGGAGGGCGATATGAAAGGTGAGGTCTATCCATTTGGCATCGTTGGGATGGCCAACAAAGGGGATT |
| | GCCTGCAGAAAGGGGAGAGCGTCAAGTTCCAATTGTGTGTCCTGGGCCAAAATGCACAAACTATGGCTTA |
| | CAACATCACACCCCTGCGCAGGGCCACAGTGGAATGTGTGAAAGATCAGTTTGGCTTCATTAACTATGAA |
| | GTAGGAGATAGCAAGAAGCTCTTTTTCCATGTGAAAGAAGTTCAGGATGGCATTGAGCTACAGGCAGGAG |
| | ATGAGGTGGAGTTCTCAGTGATTCTTAATCAGCGCACTGGCAAGTGCAGCGCCTGTAATGTTTGGCGAGT |
| | CTGTGAGGGCCCCAAGGCTGTTGCAGCTCCTCGACCTGATCGGTTGGTCAATCGCTTGAAGAATATCACT |
| | CTGGATGATGCCAGTGCTCCTCGCCTAATGGTTCTTCGTCAGCCAAGGGGACCAGATAACTCAATGGGGT |
| | TTGGTGCAGAAAGAAAGATCCGTCAAGCTGGTGTCATTGACTAACCACATCCACAAAGCACACCATTAAT |
| | CCACTATGATCAAGTTGGGGGGAATCTGGTGAAGGGTTCTGAATATCTCCCTCTTCATCCCTCCCGAAAT |
| | CTGGAATACTTATTCTATTGAGCTATTACACCAGTTTTAACACCTTCCTCGTGTTATGTTTAAAAAAATA |
| | AATAAATTTAAGAAAACCATTTTAAATAATGCACAGTTGCAGCCTGGAAAAACTTAAGGTGGCGCCTTAT |
| | AGTATCAATTTTAGGAGCTTTATTTGGTGCATTTAACGCAACTGGTAATTGCAGAATCCACTTTGCCTGT |
| | GTAAGTGAAAAATATAGACTGTTATCTTGTTGGCCCTATGAAATTCTGCACTTTTCATTATATACTCTAC |
| | CTTCATTAATTACTTCTGGCAAGATGTTCTGCCTTAGCACTCAGTTGCATTCTTTTCCTTTTTCTTCCTG |
| | TTCATTATGCTTTAATTCTGAGGACCATATGAGGGTAGAATATATTATCTTTTAAAAATTACAAAAATTT |
| | GTATAGGCAAACCATTTCTTAAAGTTGATGGCCAAATTTTAAAATGTTATTTTTCATATCATTTATAATC |
| | TTGTCACAATCCACTTAAAGAAGTTTGGTTATATTTCAGTGAAAATTTTCTTCCAGAGTAGGTTTTTTTT |
| | CGTGGGTTGGGGGGTAACTTTACTACAATTAGTAAGTATGGTGCAGAATTTCATGCAAATGAGGAGTGCC |
| | AGCAGTGTGATAATTTAAACATATTTAAACAAAAACAAAAAAAATGAATGCACAAACTTGCTGCTGCTTA |
| | GATCACTGCAGCTTCTAGGACCCGGTTTCTTTTACTGATTTAAAAACAAAACAAAAAAAAATAAAAAAGT |
| | TGTGCCTGAAATGAATCTTGTTTTTTTTTATAAGTAGCCGCCTGGTTACTGTGTCCTGTAAAATACAGAC |
| | ACTTGACCCTTGGTGTAGCTTCTGTTCAACTTTATATCACGGGAATGGATGGGTCTGATTTCTTGGCCCT |
| | CTTCTTGAATTGGCCATATACAGGGTCCCTGGCCAGTGGACTGAAGGCTTTGTCTAAGATGACAAGGGTC |
| | AGCTCAGGGGATGTGGGGGAGGGCGGTTTTATCTTCCCCCTTGTCGTTTGAGGTTTTGATCTCTGGGTAA |
| | AGAGGCCGTTTATCTTTGTAAACACGAAACATTTTTGCTTTCTCCAGTTTTCTGTTAATGGCGAAAGAAT |
| | GGAAGCGAATAAAGTTTACTGATTTTTGAGACACTA |
